# Analysis of the Interventions of Medical Emergency Teams in Older Patients in Selected Polish Cities with County Status: A Retrospective Cohort Study

**DOI:** 10.3390/ijerph18147664

**Published:** 2021-07-19

**Authors:** Mariusz Celiński, Mateusz Cybulski, Joanna Fiłon, Marta Muszalik, Mariusz Goniewicz, Elżbieta Krajewska-Kułak, Anna Ślifirczyk

**Affiliations:** 1 Department of Emergency Medicine, Faculty of Health Sciences, Pope John Paul II State School of Higher Education in Biała Podlaska, 21-500 Biała Podlaska, Poland; manieek@poczta.onet.pl (M.C.); aslifirczyk1@gmail.com (A.Ś.); 2 Department of Integrated Medical Care, Faculty of Health Sciences, Medical University of Białystok, 15-096 Białystok, Poland; joanna.filon@umb.edu.pl (J.F.); elzbieta.krajewska-kulak@umb.edu.pl (E.K.-K.); 3 Department of Geriatrics, Faculty of Health Sciences, Collegium Medicum in Bydgoszcz, University of Nicolaus Copernicus in Toruń, 85-094 Bydgoszcz, Poland; muszalik@cm.umk.pl; 4 Interfaculty Centre for Didactics, Department of Emergency Medicine, Medical University of Lublin, 20-093 Lublin, Poland; mariusz.goniewicz@gmail.com

**Keywords:** medical emergency teams, emergency medicine, older adults, seniors, geriatrics, cardiovascular diseases, respiratory diseases, injuries

## Abstract

Introduction: Geriatric patients account for a large proportion of interventions of medical emergency teams (METs). The aim of this study was to analyse medical emergency interventions in the Biała Podlaska and Chełm (Poland) between 2016 and 2018 in a group of patients ≥ 65 years of age. Materials and Methods: We analysed medical records of 1200 older patients treated by METs in Biała Podlaska and Chełm (Lublin Province, Poland). The research was conducted from June 2019 to March 2020 at the Emergency Medical Service Station in Biała Podlaska and the Medical Rescue Station in Chełm (Independent Public Complex of Health Care Facilities). Results: A total of 92.5% of medical emergency service interventions took place at the patient’s home. The mean time of stay at the scene was 20 min. The highest number of interventions occurred between 8:00 p.m. and 8:59 p.m. There were no statistically significant differences in the type of ambulance used depending on the patient’s sex, while there was a statistically significant relationship between priority code and sex. Cardiovascular diseases were diagnosed in 40% of patients, and the symptoms were not precisely classified in almost the same percentage of patients. Mortality cases accounted for 3.1% of the 1200 interventions analysed. Ambulance dispatch resulted in the patient being transported to the hospital emergency department in 69.1% of cases. Conclusions: METs were called for a variety of diseases due to the fact that geriatric patients are not able to distinguish a life-threatening condition. Medical procedures performed by METs from Biała Podlaska and Chełm were closely related to the initial diagnoses made by these teams. It was irrelevant whether a specialist or non-specialist medical emergency service was used. Paramedics are very well trained to practice their profession and are able to provide treatment to older patients in a state of sudden life threat.

## 1. Introduction

Emergency medical service is currently one of the most important elements in the functioning of the healthcare system globally, including Poland. Modern emergency medical service attaches great importance to providing treatment to those in need at the scene. Proper organisation of tasks by the team present at the scene allows for saving the patient’s life and treating the patient in a specialist centre in accordance with medical indications. The significant increase in the older population caused a sudden increase in the demand for medical services provided by the National Medical Emergency System in Poland, and the management of a geriatric patient requires a special and individual approach [1]. Older individuals are one of the groups with the highest rates of emergency department (ED) visits [2]. Challenges that impact ED care for this patient population include multiple morbidities, atypical symptoms, polypharmacy, and drug interactions, as well as misuse of prescription and over-the-counter medications [3,4,5]. Additionally, older adults may present with functional disabilities, impaired cognition, and communication problems [6,7]. The most common health problems in adults over 65 years of age who use emergency medical care include cardiovascular (CV) disorders, such as ischaemic heart disease, directly contributing to myocardial infarction, which carry the risk of stroke and cause hypertensive disease. Furthermore, medical emergencies faced by METs include respiratory failure (asthma, COPD) and injuries resulting from falls and subsequent complications during convalescence [8].

The main aim of this study was to assess METs interventions in adults ≥ 65 years of age in Biała Podlaska and Chełm between 2016 and 2018. We attempted to verify the preliminary diagnosis and actions taken by medical emergency teams at the scene of incidents, and thus the initial diagnosis made, as well as an attempt to characterise the clinical entities for which METs were called, as well as the types of ambulance (i.e., specialist or basic ambulance) sent to the scene. The following research hypotheses were formulated:

1. The most common clinical entities requiring emergency services include CV diseases, injuries due to external causes and respiratory diseases.
2. Basic METs are more often sent to geriatric patients.

## 2. Materials and Methods

### 2.1. Research Material

We analysed medical records of 1200 older patients (≥65 years) treated by METs from Biała Podlaska (600 sheets of medical records) and Chełm (600 sheets of medical records) in the Lublin Province (Poland). Although the emergency request forms were selected randomly, age ≥ 65 years was the main inclusion criterion. We analysed 2016–2018 data. We analysed 400 sheets of medical records in a year (200 sheets from one county). The analyses covered 12 consecutive months of the year, which provided about 17 forms per month.

### 2.2. Research Methods

The research was conducted from June 2019 to March 2020 at the Emergency Medical Service Station in Biała Podlaska and the Medical Rescue Station in Chełm. Data collection was based on the analysis of medical documentation, i.e., medical emergency service request forms and emergency medical procedure forms, which are stored in the Emergency Medical Service Station in Biała Podlaska and the Medical Rescue Station in Chełm. These two documents were integrated and were the only documentation of METs services. The obligation to use these forms is regulated by the Regulation of the Minister of Health of on the types and scope of medical documentation and the manner in which it is processed dated 21 December 2010. The emergency medical procedure form may be electronic or paper; however, it is always issued in two copies, one of which is given to the patient or their legal representative, and, in the case of transporting the patient to the hospital, it is handed in to the doctor on duty in the Hospital Emergency Department on a given day. Therefore, it is a medical documentation for the patient (if not transported to the hospital) or for HEDs personnel, and may be used as evidence in prosecutor’s proceedings; therefore, it must be carefully completed in each case [9].

In order to be able to achieve the set goals, we obtained prior consents for conducting the study from the heads of emergency medical institutions.

After submission of the application and receiving approval from the Bioethics Committee of the Medical University of Białystok (approval no. R-I-002/26/2019 of 31 January 2019), we analysed medical documents.

Medical records were analysed for the reason of MET activation by the medical dispatcher, who, after a prior conversation with the caller, requested a MET to leave, thus specifying the priority code of the medical team’s departure to the scene, i.e., light and siren (high priority) or without light or siren (standard). The main task of a medical dispatcher was also to determine the place of the incident, i.e., where a given person expected help (at home, in a public place, in the street, at work, at school, or in agriculture), as well as arranging an appropriate medical team: basic ambulance (without a doctor) or a specialist ambulance (with a doctor).

All these activities were recorded by the medical dispatcher in the request form under the “Acceptance of the call” and “Decision” sections. Medical records were additionally analysed in terms of the time of receipt of the call by the medical dispatcher, as well as the time of reaching the patient, the length of stay at the scene, and the implementation of medical procedures by the paramedics. The time of handing the patient over to the doctor on duty at the HEDs was another important time parameter. It should be noted at this point that this time was not recorded in all patients, which was caused by the team’s decision to leave the patient at home (at the scene). The total time of intervention was the last parameter assessed. All the above data were reported by the head of the medical team in the “Order execution” section.

Furthermore, we analysed medical emergency activities after the arrival at the scene, i.e., blood pressure, pulse, respiration, saturation, and glucose measurements, as well as the assessment of the level of consciousness based on the Glasgow Coma Scale (GCS). Additional measures performed by the team included heart rate measurements (ECG), insertion of intravascular access, administration of drugs and/or oxygen, temperature measurements, dressing and immobilisation, and direct cardiac massage with defibrillation, as well as intubation of the upper respiratory tract. The team also secured patients with a spine board and a cervical collar, as well as checking the patients for alcohol consumption. All performed activities were recorded by the team’s head in the emergency medical procedure form under the “Examination” and “Patient Management” sections. The team’s head was also obliged to make a diagnosis in a given older patient after entering vital parameters in the “Diagnosis” section.

### 2.3. Statistical Analysis

In the descriptive part, the characteristics of the studied population was performed in the form of tables containing the percentage distribution of selected features or the values of selected descriptive statistics for numerical features. The following numerical characteristics of the parameters studied were most often estimated: arithmetic mean, median (middle value), the maximum and the minimum value, standard deviation (SD), and upper (*c_75_*) and lower (*c_25_*) quartile.

The verification of more complex research hypotheses required an analysis of the correlations between various features, and the selection of a statistical method depended on the nature of the compared parameters.

If both features were nominal (text), we compared the percentage distribution of variants of one feature in the compared groups and assessed the significance of differences between them using the chi-squared test of independence.

The analysis of the relationship between a nominal feature (e.g., the mode of call or the diagnosis made by the METs) and a numerical variable (e.g., patient’s age or time of intervention) consisted in comparing the values of descriptive statistics of the numerical feature in the compared groups. The significance of differences in the distribution of a numerical feature between the compared groups was assessed using the Mann–Whitney *U* test (StatSoft Company, Hamburg, Germany) for two groups and the Kruskal–Wallis test (StatSoft Company, Hamburg, Germany) for three or more groups.

The data were supplemented with the results of the significance test of the correlation coefficient (*p*), which made it possible to assess whether the relationship found in the sample reflected a more general relationship in the entire population, or whether it was only incidental.

The level of statistical significance was set at *p* < 0.05, where

1. for *p* > 0.05, there is no reason to reject the null hypothesis, which means that the tested difference, relationship, or effect is not statistically significant;
2. for *p* < 0.05, there is a statistically significant relationship (*);
3. *p* < 0.01 indicates a highly statistically significant relationship (**);
4. *p* < 0.001 indicates an extremely highly statistically significant relationship (***).

## 3. Results

### 3.1. General Demographics

The socio-demographic characteristics of patients whose medical records were analysed are shown in Table 1. Women accounted for the majority of the study population (almost 60% of all analysed cases of emergency interventions). The number of urban and rural patients was almost identical, which was not due to the deliberate selection of the sample. People with vocational education dominated in the study group of patients using emergency services, accounting for almost 50% of all patients. Although the study population included older adults, almost half of them were still married. Slightly less than half (42%) were widowed. Single and divorced patients accounted for 10% of the study group (Table 1).

The mean age of patients managed by METs was 77 years (SD of about 8 years). One in four patients was ≤70 years and ≥84 years of age. After a subdivision into 5-year age groups, patients aged 65–69 years and 80–84 years dominated. The age structure of patients using emergency medical services is also shown by sex, assuming quite significant age differences between patients of both sexes, which resulted from demographic conditions. The age comparison of women and men was performed using descriptive statistics. Female patients were older by an average of about 3.5 years (the difference was 6 years for comparison of median values) (Table 2).

### 3.2. Data on METs Interventions

The investigated population was homogeneous in terms of the scene of incident, with more than 90% of interventions taking place in patient’s home (in women significantly more often than in men). Therefore, this factor was not taken into account in further analyses. In slightly more than 60% of cases, a basic ambulance was called, whereas an ambulance with lights and siren was needed in one-third of cases (significantly more often among women). This was due to a higher number of basic ambulances than specialist ones. Almost all patients underwent standard medical procedures: GCS (Glasgow Coma Scale) to assess the level of consciousness, checking for breathing, and non-invasive blood pressure (NIBP) measurement. Peripheral oxygen saturation (SpO_2_) was measured in about three-quarters of patients. The remaining procedures were performed in less than half of the patients, with the most common ones including ECG, pharmacotherapy, and blood glucose measurement. Such a percentage distribution of medical procedures performed by METs resulted from the patient’s medical condition and the individual approach to the clinical picture in a given patient. Medical procedures such as immobilisation and spine board were used significantly more often in women, while in men, they were used significantly more often catheter and oxygen (Table 3).

Handing the patient over to an ED physician was reported in 829 cases (69% of all interventions). The time of reaching the patient and transporting the patient to ED depended on where the incidence took place. The length of stay at patient’s home (or at the scene if the event did not occur at patient’s home) was the most objective measure, which did not depend on the location of the scene. The mean length of stay at the scene was 20 min and was at least 25 min in one in four patients (Table 4). The lowest number of interventions occurred at night (between 11 p.m. and 4:59 a.m.). The daily peak was between 8:00 p.m. and 8:59 p.m.; however, the distribution of the number of interventions during the day was generally homogeneous.

### 3.3. Demographic Factors and MET Interventions

There were no statistically significant differences in the type of ambulance used depending on the patient’s sex (*p* > 0.05). A statistically significant (*p* = 0.0001 ***) relationship was found between sex and priority code. Light and siren (L&S) interventions were more common among women (30.2 vs. 41.0%).

It is not possible to confirm the impact of the patient’s age on the priority code in the group of men (*p* = 0.9074)—the percentage of high-priority (L&S) interventions ranged from 34.6% to 44.8% in the individual age groups. However, there were statistically significant differences among women; however, the relationship was not very easy to interpret, because while the high percentage (47.5%) of high-priority (L&S) interventions in the group of women aged ≥90 years can be easily explained, the fact that it was also higher than in the remaining groups in the youngest age group (65–69 years—34.2%) is more difficult to interpret (Table 5).

There were no statistically significant differences between the age and the length of stay at the scene and between the age and the duration of MET interventions. The correlations were statistically insignificant in both women and men (*p* > 0.05).

### 3.4. Relationships between the Selected Features of MET Interventions

Almost half of specialist ambulance trips and only one in four basic ambulance trips used warning lights and sirens (Table 6).

There was no statistically significant relationship between the priority code and the length of stay at the scene (*p* > 0.05). The length of stay at the scene depending on the type of ambulance was also assessed in a similar way. Again, no differences were found. The average length of stay at the scene was almost identical for both types of ambulances (the difference of about 1 min was of no importance for the functioning of the emergency medical services). We compared the length of stay at the scene depending on the decision on admission to ED also. In the case of admission, the length of stay was on average 12 min shorter (nearly two times shorter) than when no admission was made. The difference was statistically significant (*p* = 0.0000 ***) (Table 7).

### 3.5. Diagnosis Made by METs

The diagnoses classified on the basis of ICD–10 chapters are presented in the table below (beginning with the most common ones).

Cardiovascular diseases were diagnosed in 40% of patients, and the symptoms were not precisely classified in almost the same percentage of patients. Other diseases were less common, with the most common ones including injuries, poisoning, and other external factors, which were reported in 12.6% of all patients (Table 8). Mortality cases accounted for 3.1% (one in 30 patients) of the 1200 interventions analysed.

### 3.6. Further Patient Management by METs

As may be seen in the Table 9, two-thirds of ambulance trips resulted in patient transportation to ED in a specialist hospital for detailed diagnosis.

ED admissions were more common among men than women, but not significant. It may be therefore concluded that there was no relationship between patient’s sex and the risk of the need for admission to ED.

However, there was a significant relationship between the need for admission to ED and patient’s age (*p* = 0.0034 **). Surprisingly, there was found that patients ≥90 years of age were less frequently transported to ED than their younger counterparts (Table 9).

The use of specialised ambulance reduced the risk of admission to ED (63%) as opposed to a basic ambulance (73%). There was no correlation between further METs management and the fact whether it was L&S or standard intervention. About 70% of patients were admitted to the Hospital Emergency Department (HED) (Table 10).

## 4. Discussion

The medical emergency system has dynamically evolved in recent years and has become a subject of an increasing number of scientific papers, whose main aim is an attempt to reasonably improve the functioning of emergency teams in Poland. Thus far, the model of the National Medical Emergency has not been thoroughly analysed, especially in terms of the role of specialist and basic teams, as well as the level of using qualifications of METs doctors and paramedics [10].

In our study, basic ambulances were used in 65.3% (784) of MET interventions. In other studies [11,12,13] conducted in Poland, this tendency was confirmed, while the share of basic METs were slightly higher than in our study (68.66−74.72%). The similarity of these findings results from the fact that teams without a doctor are a more widespread than ambulances with a doctor. This is due to the fact that for several years there has been a downward trend in the number of specialist teams throughout Poland. To a large extent, this is caused by the conscious action of state authorities, as well as the heads of individual medical emergency institutions in consultation with provincial governors, who increasingly consider that medical action should be taken by doctors only at the hospital level, and the pre-hospital treatment should be performed by paramedics and nurses, who are increasingly well-trained, and whose role in the medical emergency system is increasing, extending the range of medical emergency activities that can be undertaken at the scene of event.

Our analysis of dispatching ambulances with priority no L&S code showed that 65.3% of all trips were urgent. In other Polish studies [12,13,14], this tendency was also confirmed, while the number of non-priority interventions was higher than in our study (72.7–88.1%). Different findings were obtained by Filip et al., who showed that nearly all (90.6%) of interventions were high priority (L&S) [11]. Moreover, in this case, the convergence of research results is almost uniform. This is due to the fact that METs are sent to almost every clinical entity, and most of the diseases reported by patients do not require the use of lights or siren, and thus do not expose medical personnel to additional danger in road traffic. Only in the study by Filip et al. were almost all interventions coded as high priority. Such a discrepancy may have been due to the willingness of the dispatchers of METs to reach the patient as soon as possible, without exceeding the median time from the moment of receiving the call to reaching the patient by METs, which is 8 min in urban areas, and should not exceed 15 min outside the city.

Referring to the above, the average time of reaching the patient in our study was 8 min. The average length of stay at the scene was 20 min, and it was not less than 25 min in one in four patients. Kózka et al. showed that the mean (median) time of reaching the patient was 7 min, and the time of stay at the scene was not longer than 22 min [15]. The action plan of the State Medical Rescue System for the Podkarpackie Voivodeship and the analysis contained therein also reports the time of reaching the scene by a medical emergency service team. The time of reaching the patient was at least one minute and up to 85 min for the entire operating area. Most often, METs reached the scene in 7 min (9.57%); the median time of reaching the scene was 9 min in 2011, and the upper quartile was 15 min [16]. According to the German Resuscitation Registry, EMS teams reached the patient within 8 min of the call [17]. Leonard et al. [18] found that the mean time of reaching the scene was 7 min for urban agglomeration and 16 min outside the city. The mean length of stay at the scene was 17 min. The mean time of reaching the scene is similar. To a large extent, this is imposed by the State Medical Emergency System itself, which is aimed at improving the work of medical emergency services, forcing the dispatchers to deploy METs in such a way that they can quickly and safely reach a person in need of specialist medical care [19]. It can also be presumed that the duration of the METs stay may be longer in older patients. This is most often due to the fact that geriatric patients have more comorbidities. Thus, METs need relatively more time to make a correct diagnosis and decide whether to transport the patient to HEDs.

Analysing the daily cycle of MET interventions in our study, we found that the lowest number of interventions took place at night, and most of METs interventions were carried out in the morning and during the day, which seems to be confirmed by the above-mentioned studies [9,20,21,22]. In our study, the daily peak was between 8:00 p.m. and 8:59 p.m., but the distribution of the number of interventions during the day was generally homogeneous. The time discrepancy of the greatest concentration of interventions during the day can be explained by the fact that Polish society expects constant readiness from medical services, as people are not able to predict when their health condition will suddenly deteriorate, which can occur at any time of the day or night.

In our study, the most common medical activities performed by METs at the scene (>90% of all activities) included patient consciousness level assessment (GCS), breathing check, and blood pressure and heart rate measurements. Other authors in their studies also confirmed the performance of the same activities as the most common medical procedures performed by METs [23,24]. Moreover, in this case, the research results are convergent. This can be explained by the fact that METs operate in a strictly defined manner, performing their tasks in accordance with the standards of the European Resuscitation Council (ERC). Without checking vital parameters and performing the already limited diagnostics (i.e., ECG, NIBP, glycaemia, oxygen saturation), no MET would be able to make a correct diagnosis, which may anyway turn out to be incorrect after the patient has been transported to HEDs with a wide array of diagnostic tools.

The most common diagnoses made by METs after prior examination of the patient in our study were cardiovascular diseases; symptoms, signs, or abnormal clinical and laboratory findings, not elsewhere classified; or injuries, poisoning, or any other effects of external causes. Timler et al. found in their research that METs were most often called due to cardiovascular diseases, injuries, and respiratory diseases [21]. Gawełko and Wilk [22] showed that the most common reasons for METs calls were clinical and laboratory findings not elsewhere classified; injury, poisoning, and certain other consequences of external causes; and cardiovascular diseases The above research shows that the main reasons for METs calls among the older adults seem understandable. These include primarily cardiovascular diseases and their sequelae [25]. Injuries account for the vast majority of interventions. Making poorly detailed diagnosis under ICD-10 codes from R00 to R99 (clinical and laboratory findings, not elsewhere classified) is a problematic phenomenon in pre-hospital patient management. This is probably due to the fact that, on the one hand, medical emergency teams use this codification to report only the disease they faced, and on the other hand, they do not have tools for a broadly understood diagnosis, and therefore prefer to avoid errors by making a less detailed diagnosis. Syncope or fainting, which are included in this group of ICD-10 codes, occur in many diseases, such as neurological, cardiac, pulmonary diseases, or cancer. Only a more extensive diagnostic work-up in a hospital allows for a more detailed diagnosis. It should be noted here that an incorrect diagnosis does not result from the lack of knowledge or skills on the part of the emergency medical personnel, but only from the limited possibilities of pre-hospital care [26].

Our research focused on the issue of further METs management, i.e., transporting the patient to the HEDs. We found that ambulance trips ended with the patient being transported to an emergency department in a specialised hospital, where detailed diagnosis was initiated, in more than two-thirds of the cases. Additionally, the use of specialised ambulance reduced the risk of admission to ED (63%) as opposed to basic ambulance (73%). Jarosławska-Kolman et al. found a relatively high proportion of older adults in the general population of ED patients, which was 21.2% in 2011 up to 22.4% in the years 2012–2013 nationwide. In Warsaw, since 2011, the percentage of older people was higher than the average for Mazovia or the entire country, reaching 29.3% in 2012 [27]. The analysis by Kisiała showed that in Poland, the total number of people aged ≥65 years who received medical care from METs increased every year. The percentage of older patients in the overall population of medical service recipients ranged between 38% and 41% nationwide [28]. Carret et al. found in their research that the rates of emergency interventions among people over 60 years of age, and thus the use of ER, was 46.8%, with individuals aged 70–79 and 80–89 years accounting for the majority of patients [29]. Foreign reports also point to the international scale of the problem of excessive overcrowding of EDs. Tang et al. conducted their study in the United States, where they found that the number of annual ED admissions increased from 94.0 million to 116.8 million between 1997 and 2007, with the growing group of patients consisting of individuals aged 70–85 years (31.9%) [30]. For a comparison, Albert et al. showed that 19.6 million patients over 65 years of age were admitted to American Emergency Departments in the years 2009–2010, including 38.3% transported by METs. Injuries, falls in particular, were the most common reason for calls in the United States (13.5% of all patients ≥65 years of age) [31]. Filip et al. showed that 68.55% (15,190) of patients required admission to HEDs [11]. Therefore, we can obtain the impression that the suggestions concerning excessive use of EDs and METs are not limited to Poland. The constantly growing older population, both in terms of absolute and percentage values, as well as population changes and hitherto unresolved organisational problems require targeted action from economic and political decision-makers. When looking critically at the use of the Polish Medical Emergency System, it should be noted that a similar, so far unresolved problem is also faced by countries whose model of system solutions we adopted (Great Britain, the United States) and from whom we derive all possible standards of management in the event of the loss of homeostasis in a patient [32].

Our study has some limitations. First of all, it covered medical documentation from only two exemplary medical emergency service stations in cities with a county status, to which we had the easiest access. Therefore, the study needs to be extended in order to be representative of the entire population of older people in Poland. Secondly, the analysed group of patients is characterised by over-representation of women in relation to men. A larger (comparable) number of men should be included in future studies. Thirdly, the chosen sample does not really reflect the real case where most of the population lives in urban zones rather than rural zone.

## 5. Conclusions

1. METs were called for various diseases due to the fact that geriatric patients are not able to distinguish their life-threatening conditions.
2. Medical procedures performed by METs from Biała Podlaska and Chełm were closely related to the initial diagnoses made by these teams.
3. It was irrelevant whether medical service was carried out by specialist or basic METs. Paramedics are very well prepared to practice their profession and are able to help the older adults in a state of sudden life threat.

## Figures and Tables

**Table 1:** Socio-demographic characteristics of patients included in the analysis.

Feature		n	%
Sex	Female	702	58.5%
	Male	498	41.5%
Place of residence	Urban	592	49.3%
	Rural	608	50.7%
Education	Elementary	80	6.7%
	Vocational	589	49.1%
	Secondary	311	25.9%
	Higher	220	18.3%
Marital status	Married	566	47.2%
	Single	79	6.6%
	Divorced	50	4.2%
	Widowed	505	42.1%

**Table 2:** Age structure in the total population and by sex (a descriptive table).

Sex	Age (Years)						
	$\bar{x}$	Me	SD	*c_25_*	*c_75_*	Min	Max
Female	78.9	80	8.1	72	85	65	99
Male	75.2	74	8.2	68	82	65	101
Total	77.3	78	8.4	70	84	65	101

Abbreviations: $\bar{x}$—mean; Me—median; SD—standard deviation; *c_25_*—lower quartile; *c_75_*—upper quartile; min—minimum; max—maximum.

**Table 3:** Basic data on METs intervention.

Variable		n (%)	Men (%)	Women (%)	*p*
Scene	Home	1110 (92.5%)	449 (90.2%)	661 (94.2%)	0.0323 *
	Public place	84 (7.0%)	44 (8.8%)	40 (5.7%)	
	Street	4 (0.3%)	3 (0.6%)	1 (0.1%)	
	Agriculture	1 (0.1%)	1 (0.2%)	0 (0.0%)	
	Work	1 (0.1%)	1 (0.2%)	0 (0.0%)	
Priority code	L&S	416 (34.7%)	204 (41.0%)	212 (30.2%)	0.0001 ***
	No L&S	784 (65.3%)	294 (59.0%)	490 (69.8%)	
Type of ambulance	Basic	726 (60.5%)	288 (57.8%)	438 (62.4%)	0.1112
	Specialised	474 (39.5%)	210 (42.2%)	264 (37.6%)	
Marital status	Married	566 (47.2%)	299 (60.1%)	267 (38.0%)	0.0000 ***
	Single	79 (6.6%)	37 (7.4%)	42 (6.0%)	
	Divorced	50 (4.2%)	31 (6.2%)	19 (2.7%)	
	Widowed	505 (42.1%)	131 (26.3%)	374 (53.3%)	
Medical procedure	GCS	1173 (97.8%)	487 (97.8%)	686 (97.7%)	0.9355
	Breath	1153 (96.1%)	475 (95.4%)	678 (96.6%)	0.2912
	NIBP	1141 (95.1%)	470 (94.4%)	671 (95.6%)	0.3409
	Pulse	1131 (94.3%)	463 (93.0%)	668 (95.2%)	0.1092
	SpO_2_	894 (74.5%)	368 (73.9%)	526 (74.9%)	0.6858
	ECG	598 (49.8%)	245 (49.2%)	353 (50.3%)	0.7103
	Medications	575 (47.9%)	238 (47.8%)	337 (48.0%)	0.9416
	Glucose	549 (45.8%)	231 (46.4%)	318 (45.3%)	0.7098
	Catheter	433 (36.1%)	198 (39.8%)	235 (33.5%)	0.0255 *
	Temperature	369 (30.8%)	167 (33.5%)	202 (28.8%)	0.0784
	Oxygen	161 (13.4%)	90 (18.1%)	71 (14.3%)	0.0001 ***
	Immobilisation	76 (6.3%)	22 (4.4%)	54 (7.7%)	0.0217 *
	Dressing	73 (6.1%)	31 (6.2%)	42 (6.0%)	0.8628
	Intubation	20 (1.7%)	11 (2.2%)	9 (1.3%)	0.2166
	Cardiac massage	18 (1.5%)	9 (1.8%)	9 (1.3%)	0.4609
	Spine board	13 (1.1%)	1 (0.2%)	12 (1.7%)	0.0129 *
	Defibrillation	9 (0.8%)	6 (1.2%)	3 (0.4%)	0.1240
	Cervical collar	3 (0.3%)	0 (0.0%)	3 (0.4%)	-
	Missing data	2 (0.2%)	1 (0.2%)	1 (0.1%)	-

Abbreviations: L&S—light and siren; GCS—Glasgow Coma Scale; NIBP—non-invasive blood pressure; SpO_2_—peripheral oxygen saturation; ECG—electrocardiography; *—*p* < 0.05; ***—*p* < 0.001.

**Table 4:** Duration of MET interventions.

Duration (Hours)	Sex	$\bar{x}$	Me	SD	*c_25_*	*c_75_*	Min	Max	*p*
Reaching the patient	Overall	0:11	0:09	0:08	0:05	0:16	0:01	1:01	0.2287
	Men	0:11	0:08	0:08	0:06	0:16	0:01	0:43	
	Women	0:12	0:10	0:09	0:05	0:16	0:01	1:01	
Stay at the scene	Overall	0:20	0:17	0:11	0:12	0:25	0:03	1:40	0.5134
	Men	0:20	0:17	0:12	0:12	0:24	0:03	1:40	
	Women	0:20	0:17	0:12	0:12	0:25	0:04	1:22	
From taking action to handing over to ED	Overall ^1^	0:33	0:30	0:15	0:23	0:40	0:09	1:50	0.3093
	Men ^2^	0:33	0:30	0:15	0:23	0:38	0:09	1:50	
	Women ^3^	0:34	0:30	0:15	0:23	0:40	0:11	1:45	
Entire intervention	Overall	1:00	0:56	0:25	0:43	1:14	0:16	3:08	0.4749
	Men	1:01	0:55	0:35	0:43	1:11	0:18	2:43	
	Women	1:02	0:56	0:27	0:43	1:17	0:16	3:08	

^1^ Concerns 829 patients who were transferred to the HEDs. ^2^ Concerns 361 men who were transferred to the HEDs. ^3^ Concerns 468 women who were transferred to the HEDs. Abbreviations: $\bar{x}$—mean; Me—median; SD—standard deviation; *c_25_*—lower quartile; *c_75_*—upper quartile; min—minimum; max—maximum; ED—emergency department; HED—hospital emergency department.

**Table 5:** Priority code for intervention depending on age and sex.

Age Group (Years)	Sex			
	Females		Males	
	Priority Code (*p* = 0.0260 *)		Priority Code (*p* = 0.9074)	
	L&S	No L&S	L&S	No L&S
65–69	40 (34.2%)	77 (65.8%)	65 (40.6%)	95 (59.4%)
70–74	30 (28.0%)	77 (72.0%)	43 (44.8%)	53 (55.2%)
75–79	34 (27.0%)	92 (73.0%)	32 (42.7%)	43 (57.3%)
80–84	46 (29.7%)	109 (70.3%)	35 (37.6%)	58 (62.4%)
85–89	33 (24.3%)	103 (75.7%)	20 (41.7%)	28 (58.3%)
90+	29 (47.5%)	32 (52.5%)	9 (34.6%)	17 (65.4%)

Abbreviation: L&S—light and siren; *—*p* < 0.05.

**Table 6:** A relationship between the type of ambulance and the priority code.

Priority Code	Type of Ambulance (*p* = 0.0000 ***)		Total
	Basic	Specialist	
L&S	194 (26.7%)	222 (46.8%)	416
No L&S	532 (73.3%)	252 (53.2%)	784
Total	726	474	1200

Abbreviation: L&S—light and siren; ***—*p* < 0.001.

**Table 7:** The impact of priority code and the length of stay at the scene, the type of METs and the length of stay at the scene, and the length of stay at the scene of event depending on the decision to transport the patient to ED.

Item		Length of Stay at the Scene							*p*
		$\bar{x}$	Me	SD	*c_25_*	*c_75_*	Min	Max	
Transport to ED	No	0:28	0:25	0:13	0:19	0:35	0:05	1:22	0.0000 ***
	Yes	0:16	0:14	0:08	0:11	0:20	0:03	1:40	
Priority code	L&S	0:20	0:17	0:12	0:12	0:25	0:04	1:22	0.7656
	No L&S	0:19	0:17	0:11	0:12	0:25	0:03	1:40	
Type of ambulance	Basic	0:19	0:17	0:10	0:12	0:24	0:03	1:21	0.8598
	Specialised	0:20	0:16	0:13	0:12	0:26	0:03	1:40	

Abbreviations: $\bar{x}$—mean; Me—median; SD—standard deviation; *c_25_*—lower quartile; *c_75_*—upper quartile; min—minimum; max—maximum; L&S—light and siren; ***—*p* < 0.001.

**Table 8:** Percentage distribution of disease incidence in geriatric patients.

Diagnosis Made by the METs Head—ICD-10 Chapters	n	% ^1^
IX. Cardiovascular diseases (I00-I99)	482	40.2%
XVIII. Clinical and laboratory findings, not elsewhere classified (R00-R99)	452	37.7%
XIX. Injury, poisoning, and certain other consequences of external causes (S00-T98)	151	12.6%
X. Diseases of the respiratory system (J00-J99)	56	4.7%
IV. Endocrine, nutritional, and metabolic diseases (E00-E90)	41	3.4%
V. Mental and behavioural disorders (F00-F99)	31	2.6%
II. Cancer (C00-D48)	24	2.0%
VI. Diseases of the nervous system (G00-G99)	23	1.9%
XIV. Diseases of the genitourinary system (N00-N99)	22	1.8%
XX. External causes of morbidity and mortality (V01-Y98)	18	1.5%
XXI. Factors influencing health status and contact with health services (Z00-Z99)	16	1.3%
XI. Diseases of the digestive system (K00-K93)	12	1.0%
XIII. Diseases of the musculoskeletal system and connective tissue (M00-M99)	8	0.7%
I. Certain infectious and parasitic diseases (A00-B99)	2	0.2%
III. Diseases of the blood and blood-forming organs and certain disorders involving the immune mechanism (D50-D89)	2	0.2%
XII. Diseases of the skin and subcutaneous tissue (L00-L99)	2	0.2%

^1^ The sum does not have to be 100%, as more than one diagnosis may have been made in one patient. Abbreviations: METs—medical emergency teams; ICD-10—International Statistical Classification of Diseases and Related Health Problems.

**Table 9:** The number of patients transported by METs to EDs by patient’s age.

Age Group (Years)	Admission to ED (*p* = 0.0034 **)		Total
	No	Yes	
65–69	78 (28.2%)	199 (71.8%)	277
70–74	61 (30.0%)	142 (70.0%)	203
75–79	59 (29.4%)	142 (70.6%)	201
80–84	77 (31.0%)	171 (69.0%)	248
85–89	52 (28.3%)	132 (71.7%)	184
90+	44 (50.6%)	43 (49.4%)	87
Total	371	829	1200

Abbreviation: ED—emergency department; **—*p* < 0.01.

**Table 10:** The number of patients transported by METs to ED by ambulance type.

Admission to ED	Type of Ambulance (*p* = 0.0001 ***)		Priority Code (*p* = 0.8323)		Total
	Basic	Specialist	L&S	No L&S	
No	194 (26.7%)	177 (37.3%)	127 (30.5%)	244 (31.1%)	371
Yes	532 (73.3%)	297 (62.7%)	289 (69.5%)	540 (68.9%)	829
Total	726	474	416	784	1200

Abbreviations: ED—emergency department; L&S—light and siren; ***—*p* < 0.001.

## Data Availability

Data are available upon reasonable request.
